# Implication of metabolic and dopamine transporter PET in dementia with Lewy bodies

**DOI:** 10.1038/s41598-021-93442-y

**Published:** 2021-07-13

**Authors:** Sung Woo Kang, Seun Jeon, Young-gun Lee, Mincheol Park, Kyoungwon Baik, Jin Ho Jung, Seok Jong Chung, Han Soo Yoo, Seong Ho Jeong, Mijin Yun, Phil Hyu Lee, Young H. Sohn, Alan C. Evans, Byoung Seok Ye

**Affiliations:** 1 Department of Neurology, Yonsei University College of Medicine, 50-1 Yonsei-ro, Seodaemun-gu, Seoul, 03722 South Korea; 2 Brain Research Institute, Yonsei University College of Medicine, Seoul, Korea; 3 Department of Neurology, Yongin Severance Hospital, Yonsei University Health System, Yongin, Korea; 4 Department of Nuclear Medicine, Yonsei University College of Medicine, Seoul, Korea; 5 Department of Neurology, Inje University Sanggye Paik Hospital, Seoul, Korea; 6 McGill Centre for Integrative Neuroscience, Montreal Neurological Institute, McGill University, Montreal, Canada

**Keywords:** Dementia, Neurodegenerative diseases

## Abstract

To evaluate the implication of ^18^F-fluorodeoxyglucose (FDG)- and dopamine transporter (DAT)-positron emission tomography (PET) in the diagnosis and clinical symptoms of dementia with Lewy bodies (DLB), 55 DLB patients and 49 controls underwent neuropsychological evaluation and FDG-, DAT-, and ^18^F-Florbetaben (FBB) PET. DAT- and FDG-uptake and FDG/DAT ratio were measured in the anterior and posterior striatum. The first principal component (PC1) of FDG subject residual profiles was identified for each subject. Receiver operating characteristic curve analyses for the diagnosis of DLB were performed using FDG- and DAT-PET biomarkers as predictors, and general linear models for motor severity and cognitive scores were performed adding FBB standardized uptake value ratio as a predictor. Increased metabolism in the bilateral putamen, vermis, and somato-motor cortices, which characterized PC1, was observed in the DLB group, compared to the control group. A combination of posterior putamen FDG/DAT ratio and PC1 showed the highest diagnostic accuracy (91.8% sensitivity and 96.4% specificity), which was significantly greater than that obtained by DAT uptake alone. Striatal DAT uptake and PC1 independently contributed to motor severity and language, memory, frontal/executive, and general cognitive dysfunction in DLB patients, while only PC1 contributed to attention and visuospatial dysfunction.

## Introduction

Dementia with Lewy bodies (DLB) is the second most common cause of dementia. However, the diagnostic sensitivity for DLB in clinical practice is suboptimal due to the absence of direct biomarkers for α-synuclein, and the high co-incidence rate or clinical overlap with other dementia-causing conditions such as Alzheimer’s disease (AD)^1^. Although reduced dopamine transporter (DAT) uptake on DAT positron emission tomography (PET) is a useful imaging biomarker for the differential diagnosis of DLB from AD^2^, the sensitivity of DAT imaging is relatively lower than its specificity. Furthermore, its diagnostic performance has not been evaluated in the distinction between DLB and healthy aging.

Metabolic changes seen on ^18^F-fluorodeoxyglucose (FDG) PET have been reported in patients with DLB, including occipital hypometabolism^3^, relative sparing of the posterior cingulate cortex^4^, and hypermetabolism involving the basal ganglia, somato-motor cortex, and cerebellum^5^. Among these, the hypermetabolic pattern has been reported to have a negative correlation with striatal dopamine deficiency in patients with DLB^6^. In the present study, we evaluated the implications of FDG- and DAT-PET in the diagnosis of DLB and their correlation with the clinical symptoms among patients with probable DLB^7^. We hypothesized that the combination of DAT- and FDG-PET imaging has advantages over DAT-PET alone in differentiating patients with DLB from the controls, and in the explanation for the clinical symptoms of DLB.

## Methods

### Participants

The study participants comprised 63 patients clinically diagnosed with cognitive impairment due to DLB, as reported in our previous study^8^, and 49 cognitively healthy controls. Subjects underwent neurological examination, neuropsychological tests, Mini-Mental State Examination (MMSE), 3 Tesla magnetic resonance imaging (MRI), FDG-PET, DAT-PET, and ^18^F-Florbetaben (FBB) PET scans at the dementia and movement clinics of Yonsei University Severance Hospital, Seoul, Korea from November 2015 to March 2019. Ten control subjects did not undergo FBB-PET. Clinical features of DLB, including parkinsonism, rapid eye movement sleep behavior disorder (RBD), visual hallucinations, and cognitive fluctuation, were evaluated based on structured questionnaires administered by caregivers. The severity of parkinsonism was assessed according to the Movement Disorder Society’s Unified Parkinson’s Disease Rating Scale (UPDRS) motor score. The clinical diagnosis of DLB was based on the 2017 revised criteria for probable DLB^7^ and striatal DAT depletion visually assessed by a nuclear medicine expert^8^. Specifically, all subjects with DLB had at least more than two core features of DLB, except for one who had preceding dementia and one core feature of parkinsonism with an indicative biomarker of reduced DAT in the basal ganglia seen in the PET scan. As a result, all subjects with DLB meet the 2017 criteria for probable DLB. According to the 2005 criteria^9^, our DLB subjects consisted of 62 probable DLB and one possible DLB. Medication status was investigated and categorized into seven groups: antidepressants, benzodiazepines, cholinesterase inhibitors, antipsychotics, anticholinergic agents or dopamine agonists, and N-methyl-D-aspartate (NMDA) receptor antagonists.

Exclusion criteria were (1) pure vascular cognitive impairment; (2) other degenerative diseases including frontotemporal dementia, corticobasal degeneration, and progressive supranuclear palsy; (3) drug-induced cognitive impairment; and (4) other causes sufficiently explaining cognitive impairment, including epilepsy, psychiatric disorder, and structural brain lesion (e.g., tumor or hemorrhage).

### Ethics approval and consent to participate

All procedures performed in human studies were in accordance with the ethical standards of the institutional and/or national research committee and with the 1964 Helsinki Declaration and its later amendments or comparable ethical standards. This study was approved by the Institutional Review Board of Yonsei University College of Medicine. Informed consent was obtained from all participants*.*

### Neuropsychological tests

All participants underwent the Seoul Neuropsychological Screening Battery^10^. Standardized z scores based on age- and education-matched norms were available for attention, language, visuospatial function, memory, and frontal/executive function. MMSE and Clinical Dementia Rating-Sum of Boxes (CDR-SOB) were measured to assess global cognition.

### Acquisition of MR images

All MRI scans were acquired using a Philips 3T scanner (Philips Intera; Philips Medical System, Best, The Netherlands) using a previously described protocol^11^.

### T1-weighted image processing

We used the FMRIB Software Library (FSL, http://www.fmrib.ox.ac.uk/fsl) for image processing. Each subject’s T1-weighted images were corrected for intensity inhomogeneity, skull-stripped, and registered to the Montreal Neurological Institute (MNI) template. The tissues in the registered images were classified into white matter, gray matter (GM), or cerebrospinal fluid (CSF) based on the hidden-Markov random field model and the associated expectation–maximization algorithm^12^. GM probability map obtained from this algorithm was non-linearly transformed into the MNI template. The striatal regions were segmented using the FMRIB’s integrated registration and segmentation tool (FIRST) algorithm, then subdivided into the anterior and posterior regions using the k-means clustering algorithm based on the voxel coordinates (Supplementary Figure 1)^13^. The striatal regions of interest (ROIs) were included in the GM class. Then, we generated a study-specific GM mask by averaging all the individual GM probability maps and binarizing the average map (> 30% GM probability), and then assigned each voxel into either background or foreground.

### Measurement of regional white matter hyperintensities (WMH)

A visual rating scale of WMH was modified from the Fazekas scale^14^. Periventricular WMH (PWMH) and deep WMH (DWMH) areas were classified according to a previously described protocol^15^.

### Acquisition, processing, and interpretation of FDG-, DAT-, and FBB-PET scans

FDG-PET, DAT-PET, and FBB-PET acquisition were performed using Discovery 600 (General Electric Healthcare, Milwaukee, MI, USA). Detailed methods for PET acquisitions have been described in a previous study^5,11^. All participants in this study underwent FDG-PET and DAT-PET. All patients with DLB and 39 out of 49 control subjects underwent FBB-PET scans.

We linearly registered FDG-PET, DAT-PET, and FBB-PET images to individual T1-weighted MRI using rigid body transformation. We performed partial volume correction within GM and white matter regions using a previously described method^16^. To generate standardized uptake value ratio (SUVR) maps for each PET modality, we used the pons, occipital cortex, and cerebellar cortex as reference regions for FDG-, DAT-, and FBB-PET in accordance with previous reports^17–20^. Then, we spatially normalized the SUVR maps to the MNI template and smoothed them using 5-mm full width at a half-maximum Gaussian kernel.

Additionally, we extracted global SUVR values from the FBB-PET as a cortical volume-weighted average of the following cortical ROIs: frontal, anterior/posterior cingulate, lateral parietal, and lateral temporal cortices. We excluded the occipital ROI in FBB-PET data analysis, as there is low β-amyloid load in AD-related changes^21^. We classified participants as β-amyloid positive or negative by applying 1.478 as global FBB-SUVR cutoff value^22^. Twenty-six of the 63 DLB patients had significant β-amyloid deposition, while four out of 39 control subjects had significant β-amyloid deposition. We identified normal cerebral metabolism in all normal control subjects. As 10–40% of cognitively normal older adults have significant amyloid deposition^23^, we did not exclude the four control subjects in our analyses to increase the generalizability of our results.

FDG/DAT ratio was calculated as representing the voxel-wise proportion of FDG and DAT uptake. Median SUVR uptakes on bilateral striatal subregions were extracted, including the bilateral anterior caudate (AC), posterior caudate (PC), anterior putamen (AP), and posterior putamen (PP). We eroded one voxel in each striatal ROIs during the extraction to minimize the partial volume effect.

We applied the scaled sub-profile model (SSM) and principal component analysis (PCA) to all subject’s three-dimensional FDG data to define a one-dimensional measure of disease progression and severity with an associated regional covariance pattern, as described in the literature^24^. Briefly, FDG SUVR maps within the study-specific GM mask were reshaped into a voxel by subject matrix. We transformed each data into logarithmic form and centered the data matrix by subtracting each subject mean and group mean voxel profile, resulting in a residual image, termed as the subject residual profile (SRP)^24^. We then applied PCA, and the reduced singular value decomposition was utilized to factorize FDG-SRP. The first principal component (PC1) was calculated for each subject and computed into the logistic regression analyses. The first component of PCA was displayed on the MNI template for visualization (Supplementary Figure 2).

To assess the difference between left and right hemispheric metabolism, we calculated the asymmetry index (AI) based on FDG-PET using the following formula:

$$ {\text{Asymmetry index }} = {\text{ 2}}00*{\text{ }}\left( {{\text{M}}_{{{\text{LH}}}} {-}{\text{ M}}_{{{\text{RH}}}} } \right)/{\text{ }}\left( {{\text{M}}_{{{\text{LH}}}}  + {\text{ M}}_{{{\text{RH}}}} } \right), $$

where M_LH_ and M_RH_ represent left and right hemispheric metabolism, respectively. To assess the degree of differences in left and right hemispheric metabolism, absolute AI was also calculated:

$$ {\text{Absolute AI }} = {\text{2}}00*{\text{ }}\left| {\left( {{\text{M}}_{{{\text{LH}}}} {-}{\text{ M}}_{{{\text{RH}}}} } \right)/{\text{ }}\left( {{\text{ M}}_{{{\text{LH}}}}  + {\text{ M}}_{{{\text{RH}}}} } \right)} \right|. $$

### Quality assurance for image processing

All MRI images and processing results were visually inspected by three researchers (SW Kang, S Jeon, and BS Ye) who were blinded to subject information for quality assurance. We excluded eight patients due to MRI motion artifacts and image processing errors in brain masking and tissue classification. Finally, 49 control subjects and 55 patients with DLB were included in the study.

### Statistical analysis

Statistical analyses for demographic and clinical data were performed using the IBM Statistical Package for the Social Sciences version 23.0 (SPSS Inc., Chicago, IL, USA). Independent t-tests and chi-square tests were performed to compare clinical features across the disease and control groups (Table 1). For AI and absolute AI, one-sample t-tests were performed to compare them with zero. Six imaging biomarkers quantitatively obtained from FDG-PET and DAT-PET were used as predictors in receiver operating characteristic (ROC) curve analyses and general linear models (GLMs), including DAT uptake in the PP (DAT-PP), AP (DAT-AP), PC (DAT-PC), and AC (DAT-AC); FDG/DAT ratio in the PP (FDR-PP); and PC1. Among bilateral DAT uptake values, lower values, rather than an average, were selected to avoid missing unilaterally abnormal DAT uptake. To avoid multi-collinearity problems, variables that had a correlation coefficient (rho) greater than 0.7 or a variance interference factor (VIF) greater than 2.5 were not simultaneously included as predictors (Table 2). Specifically, DAT-PP was highly correlated with DAT-AP, DAT-PC, DAT-AC, and FDR-PP; DAT-AP was with DAT-PC, DAT-AC, and FDR-PP; DAT-PC was with DAT-AC; and DAT-AC was with FDR-PP in overall subjects. In DLB patients, DAT-PP was highly correlated with DAT-AP, DAT-AC, and FDR-PP; DAT-AP was with DAT-PC, DAT-AC, and FDR-PP; and DAT-PC was with DAT-AC.

**Table 1:** Demographic and clinical characteristics of study participants.

	Control (n = 49)	DLB (n = 55)	*P* value
Age, year	62.4 (7.9)	74.8 (7.1)	< 0.001
Female, n (%)	26 (53.1)	23 (41.8)	0.342
Education, year	14.5 (3.9)	9.9 (5.3)	< 0.001
**Vascular risk factors, n (%)**			
HTN	12 (24.5)	27 (49.1)	0.017
DM	4 (8.2)	18 (32.7)	0.005
Dyslipidemia	13 (26.5)	20 (36.4)	0.387
**DWMH, n (%)**			0.037
Mild	40 (81.6)	34 (61.8)	
Moderate	9 (18.4)	19 (34.5)	
Severe	0	2 (3.6)	
**PWMH, n (%)**			< 0.001
Mild	40 (81.6)	21 (38.2)	
Moderate	9 (16.3)	24 (43.6)	
Severe	1 (2.0)	10 (18.2)	
**DLB features, n (%)**			NA
PARK + FLC + VH + RBD	0	10 (18.2)	
PARK + FLC + VH	0	5 (9.1)	
PARK + FLC + RBD	0	11 (20)	
PARK + VH + RBD	0	2 (3.6)	
FLC + VH + RBD	0	3 (5.5)	
PARK + FLC	0	15 (27.3)	
PARK + VH	0	1 (1.8)	
PARK + RBD	0	3 (5.5)	
FLC + VH	0	1 (1.8)	
FLC + RBD	0	3 (5.5)	
PARK	0	1 (1.8)	
UPDRS motor score	0.8 (2.9)	24.6 (13.6)	< 0.001
CDR-SOB	0.1 (0.2)	5.2 (3.9)	< 0.001
K-MMSE	29.2 (1.0)	20.9 (5.5)	< 0.001
FBB-SUVR	1.3 (0.2)	1.6 (0.4)	< 0.001
FBB-positivity, n (%)	4 (10.3)	26 (47.3)	< 0.001
Asymmetry index	− 1.2 (1.2)	− 1.6 (5.9)	0.656
Absolute asymmetry index	1.4 (1.0)	4.2 (4.3)	< 0.001
Antidepressants, n (%)	NA	15 (27.3)	NA
Benzodiazepines, n (%)	NA	13 (23.6)	NA
Cholinesterase inhibitors, n (%)	NA	24 (43.6)	NA
Antipsychotics, n (%)	NA	7 (12.7)	NA
Anticholinergic agents or dopamine agonists, n (%)	NA	10 (18.2)	NA
NMDA receptor antagonists, n (%)	NA	4 (7.3)	NA

Data are expressed in mean (SD) or number (%). Group comparisons were performed using independent *t* tests or Chi-square tests.

*CDR-SOB* clinical dementia rating sum of boxes, *DLB* dementia with Lewy bodies, *DM* diabetes mellitus, *DWMH* deep white matter hyperintensities, *FBB-SUVR* ^18^F-Florbetaben standardized uptake value ratio, *FLC* fluctuation, *HTN* hypertension, *K-MMSE* Korean version of the Mini-Mental State Examination, *NA* not applicable, *NMDA* N-methyl-d-aspartate, *PARK* parkinsonism, *PWMH* periventricular WMH, *RBD* rapid eye movement sleep behavior disorder, *UPDRS* unified Parkinson’s disease rating scale, *VH* visual hallucination.

**Table 2:** Correlation between imaging biomarkers in overall subjects and DLB patients.

	DAT-AP	DAT-PC	DAT-AC	PC1	FDR-PP	FBB-SUVR
**Overall subjects**						
*DAT-PP*						
Rho (*p* value)	0.93 (< 0.001)	0.76 (< 0.001)	0.83 (< 0.001)	− 0.52 (< 0.001)	− 0.85 (< 0.001)	− 0.14 (0.174)
VIF	7.18	2.40	3.24	1.37	3.51	1.02
*DAT-AP*						
Rho (*p* value)		0.80 (< 0.001)	0.92 (< 0.001)	− 0.54 (< 0.001)	− 0.80 (< 0.001)	− 0.14 (0.180)
VIF		2.78	6.28	1.41	2.77	1.02
*DAT-PC*						
Rho (*p* value)			0.94 (< 0.001)	− 0.65 (< 0.001)	− 0.62 (< 0.001)	− 0.18 (0.082)
VIF			8.49	1.75	1.63	1.03
*DAT-AC*						
Rho (*p* value)				− 0.67 (< 0.001)	− 0.71 (< 0.001)	− 0.21 (0.045)
VIF				1.82	1.99	1.04
*PC1*						
Rho (*p* value)					0.51 (< 0.001)	0.44 (< 0.001)
VIF					1.35	1.24
*FDR-PP*						
Rho (*p* value)						0.16 (0.135)
VIF						1.02
**DLB patients**						
*DAT-PP*						
Rho (*p* value)	0.90 (< 0.001)	0.61 (< 0.001)	0.72 (< 0.001)	− 0.28 (0.042)	− 0.86 (< 0.001)	0.10 (0.477)
VIF	5.18	1.58	2.10	1.08	3.83	1.01
*DAT-AP*						
Rho (*p* value)		0.72 (< 0.001)	0.89 (< 0.001)	− 0.43 (0.001)	− 0.80 (< 0.001)	0.07 (0.601)
VIF		2.08	4.89	1.22	2.82	1.01
*DAT-PC*						
Rho (*p* value)			0.91 (< 0.001)	− 0.40 (0.003)	− 0.50 (< 0.001)	0.11 (0.424)
VIF			5.82	1.19	1.33	1.01
*DAT-AC*						
Rho (*p* value)				− 0.51 (< 0.001)	− 0.63 (< 0.001)	0.06 (0.656)
VIF				1.34	1.65	1.00
*PC1*						
Rho (*p* value)					0.20 (0.150)	0.18 (0.199)
VIF					1.04	1.04
*FDR-PP*						
Rho (*p* value)						− 0.09 (0.510)
VIF						1.01

Rho and *p* values are results of Pearson’s correlation analyses.

*DAT* dopamine transporter, *DAT-AP* DAT uptake in the anterior putamen, *DAT-AC* DAT uptake in the anterior caudate, *DAT-PC* DAT uptake in the posterior caudate, *DAT-PP* DAT uptake in the posterior putamen, *FBB-SUVR* ^18^F-Florbetaben standardized uptake value ratio, *FDG* ^18^F-fluorodeoxyglucose uptake, *FDR-PP* FDG to DAT ratio in the posterior putamen, *PC1* the first principal component of FDG subject residual profile, *VIF* variance inflation factor.

ROC analyses were performed to find the accuracy, sensitivity, and specificity to distinguish DLB and control groups using an individual predictor or the combination of predictors. The optimal cutoff point on the ROC curve was determined using the Youden index^25^. The algorithm suggested by DeLong et al.^26^ was used to compare the area under the curves (AUCs) of individual models with the reference model that used the DAT-PP as a predictor. As FBB-SUVR was not available for 10 control subjects, we did not include FBB-SUVR as a predictor for the main ROC curve analyses. However, sensitivity analyses further including FBB-SUVR were performed (Supplementary Table 1) in which we excluded 10 control subjects with missing data from FBB-SUVR.

GLMs were used to find the effects of the predictors on UPDRS motor score, MMSE score, and standardized neuropsychological z score in DLB patients after controlling for age, sex, education, hypertension (HTN), diabetes mellitus (DM), DWMH, and PWMH. Controls were not included in these analyses, and FBB-SUVR was further included as a predictor. Predictors with a *P* value less than 0.05 on univariate analysis were then included in multivariate regression analysis. The fitness of GLMs were compared using Akaike information criterion (AIC), and the model emphasized in bold in Tables 4 and 5 had minimizing AIC. GLMs for the PC1 were performed to find the association of DAT uptake values, FDR-PP, and FBB-SUVR with the PC1. Model 1 GLMs were controlled for age, sex, education, HTN, DM, DWMH, and PWMH; Model 2 GLMs were further controlled for the UPDRS motor score from Model 1; and Model 3 GLMs were further controlled for the MMSE score from Model 1. GLMs were also performed to investigate the effects of imaging biomarkers on asymmetry indices using the same covariates.

Given the raised attention to RBD in the diagnosis of DLB, we divided our DLB patients into 23 DLB without RBD (DLB^RBD−^) and 32 DLB with RBD (DLB^RBD+^) to identify correlations of imaging biomarkers or neuropsychological test z scores with RBD. GLMs for standardized neuropsychological z scores and imaging biomarkers were used to compare the degree of cognitive dysfunctions and availabilities of imaging biomarkers across two groups after controlling for age, sex, and education.

We used the SurfStat toolbox (http://www.math.mcgill.ca/keith/surfstat/) developed in the MNI to perform voxel-wise statistical analyses. We compared FDG, FDG-SRP, DAT, and FDR between DLB and control groups using GLMs. We included age, sex, education, HTN, DM, DWMH, and PWMH as covariates. We evaluated the associations between voxel-wise FDG-SRP and DAT uptake values in the striatal ROIs (DAT-AP, DAT-PP, DAT-AC, DAT-PC, and FDR-PP) using GLMs after controlling for the same covariates. We used the false discovery rate method to correct for multiple comparisons across multiple voxels (corrected *p* < 0.05). We displayed voxel-wise statistical outcomes on MNI stereotaxic space in neurological convention.

## Results

### Demographics and clinical characteristics

The demographic and clinical characteristics of study participants are presented in Table 1. The DLB group was significantly older and less educated than the control group, and had a significantly higher prevalence of HTN and DM than the control group. There were no significant differences in terms of sex and dyslipidemia between groups. The DLB group had more severe DWMH and PWMH than the control group. Among fifty-five patients with DLB, 10 had four core features; 21 did three core features; 23 did two core features; and 1 did only parkinsonism. The DLB group had significantly higher mean UPDRS motor and CDR-SOB scores, and a lower mean MMSE score than the control group. The DLB group had a higher mean FBB-SUVR than the control group. At the time of study, there were 15 DLB patients taking antidepressants; 13 taking benzodiazepines; 24 taking cholinesterase inhibitors; seven taking antipsychotics; 10 taking anticholinergic agents or dopamine agonists; and four taking NMDA receptor antagonists. Control subjects were not taking any medications.

### ROC curve analyses

**Figure 1:**
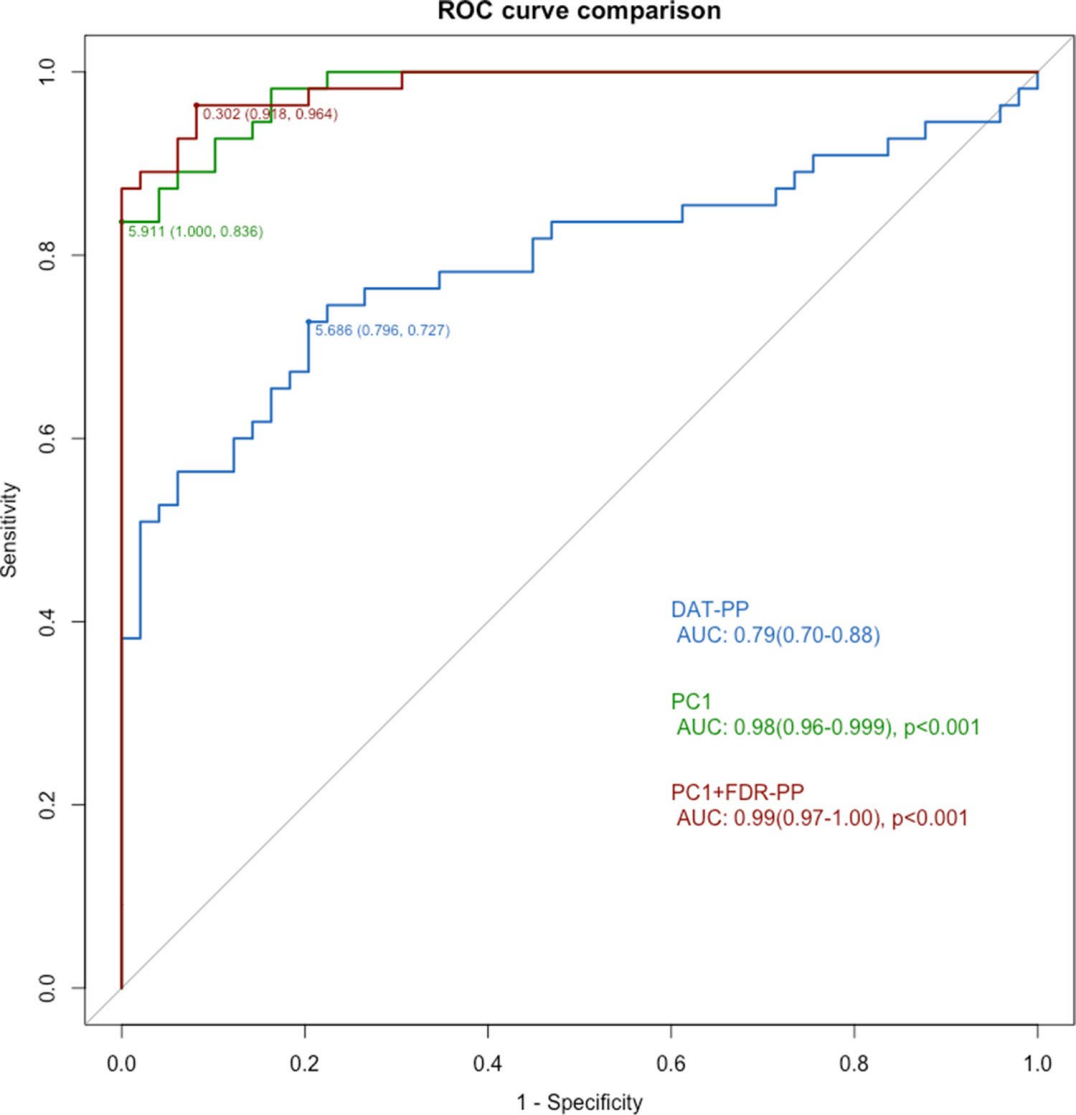
Comparison of ROC curves. DAT-PP (blue line), PC1 (green line), and PC1 + FDR-PP (red line). The numbers under each line show threshold, specificity, and sensitivity. *P* values are the result of the comparison of each model’s AUC with that of the model using DAT-PP as a predictor based on DeLong’s method. *AUC* area under the ROC curve, *DAT* dopamine transporter, *DAT-PP* DAT uptake in the posterior putamen, *FDG* ^18^F-Fluorodeoxyglucose, *FDR-PP* FDG to DAT ratio in the posterior putamen, *PC1* the first principal component of FDG subject residual profile, *ROC* receiver operating characteristic.

Table 3 shows the diagnostic accuracy of individual ROC curves ordered from lowest to highest. The ROC curve using PC1 and FDR-PP as predictors had the highest AUC (0.99, CI 0.97–1.00) (Fig. 1). The ROC curve using PC1 as a predictor and the ROC curves using the combination of PC1 with DAT-AC, DAT-PC, DAT-AP, DAT-PP, or FDR-PP as predictors had significantly higher AUCs than the reference model using DAT-PP as a sole predictor. The ROC curve using the combination of FDR-PP with DAT-PC as predictors also had significantly higher AUC than the reference model. However, models using DAT-AP, FDR-PP, DAT-AC, or DAT-PC as a predictor had AUCs comparable to the reference model. Sensitivity analyses further including FBB-SUVR as a predictor showed results that were very similar to the original results after excluding 10 control subjects who did not undergo FBB-PET (Supplementary Table 1).

**Table 3:** ROC curve analyses for the diagnosis of DLB.

Predictor	AUC (95% CI)	Threshold	Specificity (%)	Sensitivity (%)	*P* value
DAT-AP	0.75 (0.66–0.85)	0.60	87.8	56.4	0.092
DAT-PP	0.79 (0.79–0.88)	0.52	79.6	72.7	Reference
FDR-PP	0.82 (0.74–0.91)	0.59	89.8	69.1	0.225
DAT-AC	0.83 (0.75–0.91)	0.65	91.8	69.1	0.241
DAT-PC	0.85 (0.77–0.92)	0.71	93.9	65.5	0.119
DAT-PC + FDR-PP	0.88 (0.81–0.94)	0.54	85.7	81.8	< 0.001
PC1	0.98 (0.96–0.999)	0.83	100	83.6	< 0.001
DAT-PP + PC1	0.98 (0.96–0.999)	0.81	98.0	85.5	< 0.001
DAT-AP + PC1	0.98 (0.96–0.999)	0.83	100	83.6	< 0.001
DAT-PC + PC1	0.98 (0.96–0.999)	0.22	87.8	96.4	< 0.001
DAT-AC + PC1	0.98 (0.96–0.999)	0.78	95.9	87.3	< 0.001
FDR-PP + PC1	0.99 (0.97–1.00)	0.30	91.8	96.4	< 0.001

Results correspond to ROC curve analyses for the diagnosis of DLB. *P* values are the results of analyses based on DeLong’s method comparing each model’s accuracy with that of the model using DAT-PP as a predictor.

*AUC* area under the curve, *DAT* dopamine transporter, *DAT-AC* DAT uptake in the anterior caudate, *DAT-AP* DAT uptake in the anterior putamen, *DAT-PC* DAT uptake in the posterior caudate, *DAT-PP* DAT uptake in the posterior putamen, *DLB* dementia with Lewy bodies, *FDG* ^18^F-fluorodeoxyglucose uptake, *FDR-PP* FDG to DAT ratio in the posterior putamen, *PC1* the first principal component of FDG subject residual profile, *ROC* receiver operating characteristic.

### Association for imaging biomarkers with UPDRS motor scores and MMSE scores in DLB patients

Table 4 shows the associations for imaging biomarkers with UPDRS motor score and MMSE score in DLB patients. All predictors except for FBB-SUVR were significantly associated with UPDRS motor score, while DAT-PC, DAT-AC, and PC1 were significantly associated with MMSE scores in univariate models. In univariate models, DAT-AP and PC1 showed the lowest AIC values for UPDRS motor scores and MMSE scores, respectively (emphasized in bold). Multivariate regression models showed that DAT-PP, DAT-AP, DAT-PC, DAT-AC, and FDR-PP were significantly associated with UPDRS motor scores after controlling for PC1. Meanwhile, PC1 was not associated with UPDRS motor scores after controlling for DAT-AP, DAT-AC, or DAT-PC. After controlling for DAT-PP or FDR-PP, PC1 was associated with UPDRS motor scores. Multivariate regression models for MMSE score showed that PC1 and DAT-PC were independently associated with MMSE score, while DAT-AC was not, after controlling for PC1. Among multivariate models for UPDRS motor and MMSE scores, the model using DAT-AP and PC1 as predictors had the lowest AIC value for UPDRS motor score, while the model using DAT-PC and PC1 as predictors had the lowest AIC value for MMSE score.

**Table 4:** Predictors for UPDRS motor and MMSE scores in DLB patients.

		UPDRS		MMSE	
	Predictors	Beta (SE)	*P* value	Beta (SE)	*P* value
Univariate	DAT-PP	− 3.60 (1.29)	0.008	0.72 (0.55)	0.203
	DAT-AP	− **3.96 (1.13)**	**0.001**	0.98 (0.50)	0.053
	DAT-PC	− 6.31 (2.20)	0.006	2.70 (0.88)	0.004
	DAT-AC	− 4.82 (1.40)	0.001	1.86 (0.57)	0.002
	FDR-PP	36.94 (14.09)	0.012	− 6.92 (6.02)	0.256
	PC1	0.11 (0.04)	0.014	− **0.06 (0.02)**	**0.001**
	FBB-SUVR	− 5.85 (4.85)	0.234	− 1.21 (1.98)	0.544
Multivariate	DAT-PP DAT-PC	− 2.27 (1.48) − 4.19 (2.55)	0.132 0.107		
	DAT-PC FDR-PP	− 4.60 (2.44) 23.54 (15.43)	0.066 0.134		
	DAT-PP PC1	− 3.06 (1.27) 0.09 (0.04)	0.020 0.035		
	DAT-AP PC1	− **3.30 (1.18)** **0.07 (0.04)**	**0.008** **0.107**		
	DAT-PC PC1	− 5.00 (2.26) 0.08 (0.05)	0.032 0.075	**1.90 (0.85)** − **0.05 (0.02)**	**0.031** **0.006**
	DAT-AC PC1	− 3.92 (1.54) 0.06 (0.05)	0.014 0.182	1.20 (0.60) − 0.05 (0.02)	0.050 0.014
	FDR-PP DAT-PC	23.54 (15.43) − 4.60 (2.44)	0.134 0.066		
	FDR-PP DAT-AC	13.85 (16.62) − 3.94 (1.74)	0.409 0.029		
	FDR-PP PC1	33.35 (13.46) 0.10 (0.04)	0.017 0.020		

Results are based on general linear models for UPDRS motor and MMSE scores after controlling for age, sex, education, HTN, DM, DWMH, and PWMH. The model emphasized in bold reflects minimizing AICs.

*AIC* akaike information criterion, *DAT* dopamine transporter, *DAT-AC* DAT uptake in the anterior caudate, *DAT-AP* DAT uptake in the anterior putamen, *DAT-PC* DAT uptake in the posterior caudate, *DAT-PP* DAT uptake in the posterior putamen, *DLB* dementia with Lewy bodies, *DM* diabetes mellitus, *DWMH* deep white matter hyperintensities, *FBB-SUVR* ^18^F-Florbetaben standardized uptake value ratio, *FDG* ^18^F-fluorodeoxyglucose uptake, *FDR-PP* FDG to DAT ratio in the posterior putamen, *HTN* hypertension, *MMSE* mini-mental state examination, *PC1* the first principal component of FDG subject residual profile, *PWMH* periventricular WMH, *UPDRS* unified Parkinson’s disease rating scale.

### Association for imaging biomarkers with PC1 in DLB patients

DAT-AP, DAT-AC, and DAT-PC were significantly associated with PC1 in DLB patients (Supplementary Table 2). After controlling for UPDRS motor score or MMSE score, DAT-AP and DAT-PC were no longer associated with PC1, whereas the association for PC1 with DAT-AC did not change.

### Associations for imaging biomarkers with neuropsychological test scores in DLB patients

Table 5 shows the associations for imaging biomarkers with standardized neuropsychological test z scores in DLB patients. Univariate models showed that DAT-PP, DAT-AP, DAT-PC, DAT-AC, and PC1 were associated with language, memory, and frontal/executive function scores, while FDR-PP and FBB-SUVR were not significantly associated with any cognitive scores. DAT-PC, DAT-AC, and PC1 were associated with attention function score, while only PC1 was associated with visuospatial function score in univariate models. Among univariate models, the models using PC1 as a predictor had the lowest AIC values (emphasized in bold) for attention, visuospatial, memory, and frontal/executive function scores, while the model using DAT-AC did so for language function score. Multivariate models showed that PC1 and all DAT biomarkers (including DAT-PP, DAT-AP, DAT-PC, and DAT-AC) were independently associated with language, memory, and frontal/executive function scores, except the effect of DAT-AP was not significant after controlling for PC1 in memory and frontal/executive function scores. PC1 was significantly associated with attention function score after controlling for DAT-PC; however, the effect of DAT-PC was not significant after controlling for PC1. The effects of DAT-AC and PC1 on attention function score were not significant when they were simultaneously included as predictors. Among multivariate models for attention, memory, and frontal/executive function scores, the models using PC1 and DAT-PC as predictors had the lowest AIC values. Among multivariate models for language function score, the model using DAT-AC and PC1 as predictors had the lowest AIC value.

**Table 5:** Predictors for neuropsychological test scores in DLB patients.

	Predictor	Attention		Language		Visuospatial		Memory		Frontal/executive	
		Beta (SE)	*P* value	Beta (SE)	*P* value	Beta (SE)	*P* value	Beta (SE)	*P* value	Beta (SE)	*P* value
Univariate	DAT-PP	0.17 (0.11)	0.139	0.37 (0.15)	0.019	0.35 (0.34)	0.306	0.23 (0.08)	0.009	0.25 (0.10)	0.014
	DAT-AP	0.16 (0.10)	0.120	0.40 (0.14)	0.006	0.31 (0.32)	0.347	0.21 (0.08)	0.009	0.21 (0.09)	0.030
	DAT-PC	0.43 (0.20)	0.042	0.85 (0.27)	0.003	0.77 (0.64)	0.235	0.48 (0.15)	0.002	0.54 (0.16)	0.002
	DAT-AC	0.29 (0.13)	0.037	**0.62 (0.17)**	**0.001**	0.55 (0.42)	0.198	0.33 (0.10)	0.001	0.37 (0.11)	0.002
	FDR-PP	− 1.33 (1.26)	0.298	− 3.44 (1.72)	0.053	− 3.21 (3.80)	0.402	− 1.75 (0.95)	0.073	− 1.88 (1.08)	0.091
	PC1	− **0.01 (0.004)**	**0.014**	− 0.02 (0.005)	0.004	− **0.03 (0.01)**	**0.005**	− **0.01 (0.003)**	**0.001**	− **0.01 (0.003)**	**< 0.001**
	FBB-SUVR	0.31 (0.43)	0.480	− 0.07 (0.61)	0.914	1.91 (1.27)	0.140	− 0.10 (0.34)	0.775	0.15 (0.37)	0.679
Multivariate	DAT-PP DAT-PC			0.13 (0.19) 0.69 (0.36)	0.487 0.062			0.11 (0.10) 0.36 (0.19)	0.302 0.069	0.10 (0.12) 0.43 (0.21)	0.375 0.047
	DAT-PP PC1			0.30 (0.14) − 0.01 (0.005)	0.042 0.010			0.18 (0.08) − 0.01 (0.003)	0.022 0.002	0.18 (0.08) − 0.01 (0.003)	0.035 < 0.001
	DAT-AP PC1			0.31 (0.14) − 0.01 (0.005)	0.029 0.021			0.15 (0.07) − 0.01 (0.003)	0.051 0.005	0.10 (0.08) − 0.01 (0.003)	0.239 < 0.001
	DAT-PC PC1	**0.33 (0.20)** − **0.01 (0.004)**	**0.109** **0.036**	0.69 (0.26) − 0.01 (0.005)	0.011 0.015			**0.38 (0.14)** − **0.01 (0.003)**	**0.008** **0.003**	**0.39 (0.14)** − **0.01 (0.003)**	**0.009** **< 0.001**
	DAT-AC PC1	0.20 (0.14) − 0.01 (0.004)	0.158 0.057	**0.49 (0.18)** − **0.01 (0.005)**	**0.008** **0.039**			0.24 (0.10) − 0.01 (0.003)	0.015 0.009	0.23 (0.10) − 0.01 (0.003)	0.036 0.001

Results are based on general linear models for neuropsychological z scores after controlling for age, sex, education, HTN, DM, DWMH, and PWMH. The model emphasized in bold reflects minimizing AICs.

*AIC* akaike information criterion, *DAT* dopamine transporter, *DAT-AC* DAT uptake in the anterior caudate, *DAT-AP* DAT uptake in the anterior putamen, *DAT-PC* DAT uptake in the posterior caudate, *DAT-PP* DAT uptake in the posterior putamen, *DLB* dementia with Lewy bodies, *DM* diabetes mellitus, *DWMH* deep white matter hyperintensities, *FBB-SUVR* ^18^F-Florbetaben standardized uptake value ratio, *FDG* ^18^F-fluorodeoxyglucose uptake, *FDR-PP* FDG to DAT ratio in the posterior putamen, *HTN* hypertension, *PC1* the first principal component of FDG subject residual profile, *PWMH* periventricular WMH.

### Comparisons of imaging biomarkers and neuropsychological test scores between the patients with RBD (DLB^RBD+^) and those without RBD (DLB^RBD−^)

There were no significant differences in the availabilities of imaging biomarkers between DLB^RBD+^ and DLB^RBD−^ groups after controlling for age, sex, and education (Supplementary Table 3). However, the DLB^RBD+^ group exhibited better performance than the DLB^RBD−^ group in language, memory, and frontal/executive function scores. There were no significant differences in attention and visuospatial function scores.

### AI and absolute AI

Both DLB and control groups had AI values significantly lower than zero (*P* for the DLB group = 0.044 and *P* for the control group < 0.001) and absolute AI values higher than zero (*P* for the DLB group < 0.001 and *P* for the control group < 0.001) on one sample *t* tests. The DLB and control groups had comparable AI values, while the DLB group had significantly higher absolute AI value than the control group (Table 1).

GLMs for AI performed in the DLB group showed that no imaging biomarkers were associated with AI after controlling for covariates, while DAT-AC and PC1 were significantly associated with absolute AI (Supplementary Table 4). When the two predictors were simultaneously included, only PC1 had a significant effect (beta = 0.04, standard error = 0.01, *p* = 0.006), whereas DAT-AC did not (beta = − 0.36, standard error = 0.49, *p* = 0.472).

### Group comparison of voxel-wise FDG SUVR, DAT SUVR, and FDG-SRP

**Figure 2:**
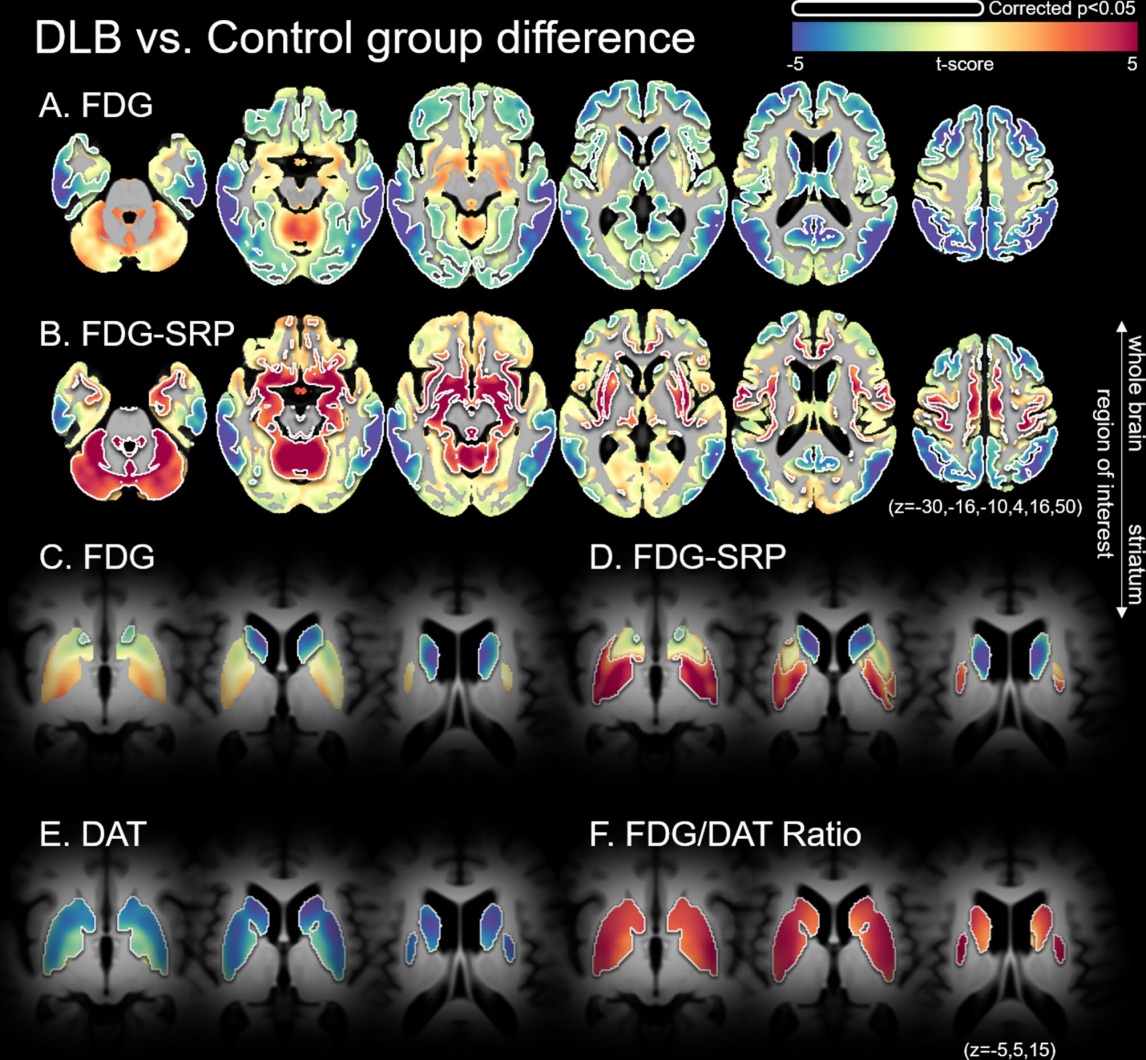
Comparison of FDG, DAT, and the FDG/DAT ratio between DLB and control groups. (**A**, **C**, **E**, **F**) are based on a general linear model for the voxel-wise standardized uptake value ratio using cerebellar crus-II as a reference region, and (**B**, **D**) are based on the general linear model for voxel-wise FDG subject residual profile. FDG/DAT ratio represents the voxel-wise proportion of FDG and DAT uptake. Age, sex, education, HTN, DM, DWMH, and PWMH are controlled for in the statistical model. The color scale indicates *t* values in the statistical analysis; red color indicates a higher metabolism in the DLB group compared to the control group, and blue color shows the inverse. Areas bounded by a white line indicate brain regions where the group difference is significant after correcting for multiple comparisons (corrected *p* < 0.05, false discovery ratio). Axial planes are displayed on Montreal Neurological Institute stereotaxic space in neurological convention (**A**, **B**: z = − 30, − 16, − 10, 4, 16, 50; **C**, **F**: z = − 5, 5, 15). *DAT* dopamine transporter, *DLB* dementia with Lewy-bodies, *DM* diabetes mellitus, *DWMH* deep white matter hyperintensities, *FDG* ^18^F-Fluorodeoxyglucose, *HTN* hypertension, *PWMH* periventricular WMH.

Compared to the control group, the DLB group had lower FDG SUVR in the bilateral caudate nuclei and widespread cortical regions, including the bilateral frontal, parietal, temporal, and occipital cortices. Although statistical significance was not achieved, the DLB group had higher FDG SUVRs in the central cerebellum, posterior putamen, and somato-motor cortex (Fig. 2). Compared to the control group, the DLB group had significantly higher FDG-SRPs in the cerebellum and limbic structures, including the hippocampus, putamen, and somato-motor cortex. The DLB group had lower FDG-SRPs in the bilateral caudate nuclei, and the bilateral lateral temporal, parietal, and frontal cortices. Compared to the control group, the DLB group had lower DAT uptake and higher FDG to DAT uptake ratio in the bilateral striatum.

### Correlation of voxel-wise FDG-SRP with imaging biomarkers

FDG-SRP in the cerebellum, limbic structure including the hippocampus, bilateral posterior putamen, and somato-motor cortex negatively correlated with DAT-AP, DAT-PP, DAT-AC, and DAT-PC, and positively correlated with FDR-PP (Supplementary Figure 3). These brain regions overlap with the increased FDG-SRP regions in the comparison between the DLB group and the control group (Fig. 2), and PC1 (Supplementary Figure 2).

## Discussion

We evaluated the performance of imaging biomarkers from FDG-PET and DAT-PET in the differentiation of patients with DLB from controls. We also evaluated the implication of these imaging biomarkers for motor features and cognitive function in DLB. Our major findings are as follows First, the spatial covariance pattern on FDG-PET (PC1), which is characterized by an increased metabolism in the bilateral posterior putamen, vermis, and somato-motor cortex, was observed in the DLB group and not in the control group. It also had better diagnostic performance than DAT-PP, which is a classic imaging biomarker for DLB. Second, the combination of PC1 with the imaging biomarker reflecting the discrepancy of FDG uptake and DAT uptake in the posterior putamen (FDR-PP) had the best diagnostic performance for DLB (AUC = 0.99, specificity = 91.8%, and sensitivity = 96.4%). Third, when DAT biomarkers and PC1 were simultaneously used as predictors in DLB patients, DAT biomarkers in the motor striatum (DAT-PP and FDR-PP) and PC1 had independent effects on UPDRS motor scores. Meanwhile, only DAT uptakes in the associative striatum (DAT-AC, DAT-PC, and DAT-AP) had significant effects on UPDRS motor scores; PC1 did not. Lastly, PC1 and striatal DAT uptake had independent effects on MMSE, language, memory, and frontal/executive function scores in DLB patients, while only PC1 was independently associated with attention and visuospatial function scores. Taken together, our results suggest that simultaneously evaluating DAT- and FDG-PET holds clinical value in the diagnosis of DLB.

Our first major finding is that PC1 had better diagnostic performance than DAT-PP. The identification of PC1 was based on data-driven multivariate methods increasingly used to examine disease-specific metabolic covariance patterns in Parkinson’s disease (PDRP)^27^, multiple system atrophy (MSARP)^28^, progressive supranuclear palsy (PSPRP)^28^, and corticobasal syndrome (CSBRP)^29^. The hypermetabolic pattern of PC1 involving the bilateral posterior putamen, vermis, and somato-motor cortex is identical to the previously known PDRP, and to the best of our knowledge, there has been no study directly comparing the diagnostic performance of PDRP and the classic biomarker of DAT-PET in the differentiation of patients with DLB from control subjects. As PC1 was identical to the previously reported PDRP^27^, our results provide evidence in support of DLB and PD as phenotypes of the LBD spectrum. Since PC1 is the most prominent metabolic pattern in our study participants, which included patients with DLB and controls, and we did not intentionally extract PC1 by comparing FDG uptake between the DLB and control groups, the utilization of PC1 in the discerning of DLB from healthy subjects is not susceptible to the circular reasoning problem^30^. Although we could not perform additional analyses for external validation due to the small sample size, our results could shed light on the breakthrough for the low diagnostic accuracy for DLB by increasing the sensitivity.

Both DLB and control groups had AI values suggesting rightward asymmetry, while the DLB group had higher absolute AI values than the control group (Table 1). These results are consistent with Chen’s previous study showing that DLB patients have significantly higher absolute AI values than controls, along with significant rightward asymmetry in terms of local and global network efficiency^31^. However, the controls in the previous study had no asymmetry in terms of global and local network efficiency, while our control group had significant rightward metabolic asymmetry. Different methodology of network analysis in the previous study could be a reason for the different laterality in our control subjects. Although the previous study showed that PD dementia (leftward asymmetry) and DLB (rightward asymmetry) patients had different asymmetric patterns in local efficiency^31^, more frequent mixed AD pathology in the DLB group than in the PD dementia group could have contributed to the different laterality^32^. Also, an increased absolute AI was shown to be characteristic of PD dementia, as well as DLB, in the previous study^31^, and PC1 well explained the increased absolute AI in our DLB patients (Supplementary Table 4). These results also suggest that DLB and PD share common pathophysiology.

Our second major finding is that the combination of FDR-PP and PC1 best differentiated patients with DLB from controls. FDR-PP reflects the discrepancy between DAT uptake and FDG uptake in the PP. DAT uptake in the striatum is decreased in PD patients^33^, but FDG uptake is preserved^34^ or relatively increased^35^. Meanwhile, both DAT uptake^36^ and FDG uptake^37^ could be decreased in patients with striatal lacunar infarction. Considering the prevalence of asymptomatic lacunar infarction in elderly people^38^, FDR-PP could have advantages over classic DAT uptake values by excluding DAT depletion due to vascular lesions. However, adding FDR-PP or DAT uptake values in any of the four striatal regions to the model using PC1 alone did not significantly increase diagnostic accuracy. Although FDR-PP was significantly associated with UPDRS motor score after controlling for PC1 in DLB patients (Table 4), PC1 provided sufficient accuracy for the diagnosis of DLB as a single biomarker (Table 3).

Our third major finding is that when DAT biomarkers and PC1 were simultaneously used as predictors, DAT biomarkers in the motor striatum (DAT-PP and FDR-PP) and DAT uptake in the associative striatum (DAT-AC, DAT-PC, and DAT-AP) consistently had significant effects on UPDRS motor scores, whereas PC1 did not, when controlling for DAT uptake in the associative striatum. As DAT uptake values were closely inter-related (Table 2), we could not compare the relative contribution of DAT uptake in the striatal subregions to clinical symptoms. However, considering that PC1 was negatively correlated with DAT uptake in the associative striatum, but not with DAT uptake in the motor striatum (Supplementary Table 2), our results seem to suggest that DAT biomarkers have more dominant effects on motor severity than PC1 in DLB patients and that DAT uptake in the associative striatum confounds the association between PC1 and motor severity score.

Lastly, PC1 and DAT biomarkers were independently associated with MMSE and language, memory, and frontal/executive function scores in DLB patients, while only PC1 was independently associated with attention and visuospatial function scores. Our results suggest that DAT biomarkers and PC1 have independent effects on cognitive dysfunction. However, considering that attention and visuospatial dysfunction, which are neuropsychological hallmarks of DLB^39^, was affected by PC1 only and that univariate models for cognitive scores using PC1 as a predictor had the lowest AIC values, PC1 could have more dominant effects on cognitive dysfunction than DAT biomarkers in DLB patients. Previous studies have shown that cognitive dysfunction in PD patients is closely related with dopaminergic depletion in the associative striatum^40–43^. Although we do not know whether metabolic changes on PC1 and dopaminergic depletion in the associative striatum have a causal relationship or there is another common underlying mechanism explaining both phenomena, dopaminergic depletion and resulting disinhibition of the basal ganglia pacemaker in the external globus pallidus-subthalamic nucleus network could be a possible explanation^44,45^.

This study has several limitations. First, the diagnosis of DLB was not made based on histopathological confirmation. However, all subjects in the DLB group were diagnosed as probable DLB and had abnormalities on DAT-PET as assessed by an expert in nuclear medicine. Second, since the control group was younger and had a longer duration of education and milder WMHs than the DLB group, caution is advised when interpreting our results. However, considering that aging^46^ and WMHs^47^ could decrease cerebral metabolism, the degree of metabolic increase in the DLB group could be underestimated by these differences. Even with these limitations, our results suggest that the relatively increased metabolism in the posterior putamen, vermis, and somato-motor cortex could be a useful imaging biomarker that could enhance the diagnostic accuracy for DLB. Furthermore, although DAT depletion and metabolic changes have more dominant effects on motor severity and cognitive dysfunction, respectively, they independently contribute to clinical symptoms of DLB patients.

## Data availability statement

The data that support the findings of this study are available from the corresponding author upon reasonable request.

## Supplementary Information

Supplementary Information.
