# Supplementary material for: Implication of metabolic and dopamine transporter PET in dementia with Lewy bodies
Source: Sci Rep. 2021 Jul 13;11:14394. doi: 10.1038/s41598-021-93442-y (PMC8277897; doi:10.1038/s41598-021-93442-y)
Supplement: Supplementary file 1 — Supplementary Information. [file 41598_2021_93442_MOESM1_ESM.docx]

**Supplementary Table 1.** ROC curve analyses for the diagnosis of DLB

| Predictor | AUC (95% CI) | Threshold | Specificity | Sensitivity | P value |
| --- | --- | --- | --- | --- | --- |
| FBB-SUVR | 0.77 (0.67-0.86) | 0.56 | 89.7% | 61.8% | 0.428 |
| DAT-AP | 0.8 (0.71-0.89) | 0.54 | 74.4% | 78.2% | 0.244 |
| DAT-PP | 0.82 (0.73-0.91) | 0.60 | 87.2% | 72.7% | Reference |
| FDR-PP | 0.84 (0.76-0.92) | 0.62 | 89.7% | 70.9% | 0.503 |
| DAT-AC | 0.86 (0.78-0.93) | 0.74 | 97.4% | 69.1% | 0.194 |
| DAT-PC | 0.87 (0.80-0.94) | 0.79 | 94.9% | 65.4% | 0.144 |
| DAT-AP + FBB-SUVR | 0.88 (0.80-0.95) | 0.58 | 87.2% | 81.8% | 0.129 |
| DAT-PP + FBB-SUVR | 0.88 (0.81-0.96) | 0.62 | 92.3% | 76.4% | 0.044 |
| DAT-PC + FDR-PP | 0.89 (0.83-0.96) | 0.64 | 87.2% | 81.8% | 0.005 |
| FDR-PP + FBB-SUVR | 0.91 (0.85-0.97) | 0.65 | 92.3% | 76.4% | 0.024 |
| DAT-AC + FBB-SUVR | 0.91 (0.85-0.97) | 0.60 | 97.4% | 81.8% | 0.011 |
| DAT-PC + FBB-SUVR | 0.93 (0.88-0.98) | 0.72 | 97.4% | 81.8% | 0.003 |
| DAT-PC + FDR-PP + FBB-SUVR | 0.94 (0.90-0.99) | 0.47 | 87.1% | 90.9% | < 0.001 |
| DAT-AP + PC1 | 0.98 (0.95-1) | 0.82 | 100% | 83.6% | < 0.001 |
| DAT-AC + PC1 | 0.98 (0.95-1) | 0.85 | 100% | 81.8% | < 0.001 |
| PC1 | 0.98 (0.95-1) | 0.83 | 100% | 83.6% | < 0.001 |
| DAT-PP + PC1 | 0.98 (0.95-1) | 0.68 | 92.3% | 90.9% | < 0.001 |
| DAT-PC + PC1 | 0.98 (0.96-1) | 0.83 | 100% | 83.6% | < 0.001 |
| DAT-AC + PC1 + FBB-SUVR | 0.98 (0.96-1) | 0.74 | 100% | 92.7% | < 0.001 |
| PC1 + FBB-SUVR | 0.98 (0.96-1) | 0.75 | 100% | 89.1% | < 0.001 |
| DAT-AP + PC1 + FBB-SUVR | 0.98 (0.96-1) | 0.74 | 100% | 90.9% | < 0.001 |
| DAT-PP + PC1 + FBB-SUVR | 0.98 (0.96-1) | 0.74 | 100% | 90.9% | < 0.001 |
| DAT-PC + PC1 + FBB-SUVR | 0.98 (0.96-1) | 0.74 | 100% | 92.7% | < 0.001 |
| FDR-PP + PC1 | 0.98 (0.97-1) | 0.75 | 100% | 89.1% | < 0.001 |
| FDR-PP + PC1 + FBB-SUVR | 0.98 (0.96-1) | 0.65 | 100% | 94.5% | < 0.001 |

Results correspond to ROC curve analyses for the diagnosis of DLB after excluding controls with missing data of global FBB SUVR (FBB-SUVR). P values are the results of analyses based on DeLong’s method comparing each model’s accuracy with that of the model using DAT-PP as a predictor.

Key: AUC, area under the curve; DAT, dopamine transporter; DAT-AC, DAT uptake in the anterior caudate; DAT-AP, DAT uptake in the anterior putamen; DAT-PC, DAT uptake in the posterior caudate; DAT-PP, DAT uptake in the posterior putamen; DLB, dementia with Lewy bodies; FBB-SUVR, ^18^F-Florbetaben standardized uptake value ratio; FDG, ^18^F-fluorodeoxyglucose; FDR-PP, FDG to DAT ratio in the posterior putamen; PC1, the first principal component of FDG subject residual profile; ROC, receiver operating characteristic.

**Supplementary Table 2.** Association for imaging biomarkers with PC1 in DLB patients

| Model | Predictor | Beta (SE) | P value |
| --- | --- | --- | --- |
| Model 1 | DAT-AP | -9.13 (3.45) | 0.008 |
|  | DAT-PP | -5.78 (3.93) | 0.141 |
|  | DAT-AC | -14.36 (4.08) | < 0.001 |
|  | DAT-PC | -16.19 (6.48) | 0.012 |
|  | FDR-PP | 35.39 (42.99) | 0.410 |
|  | FBB-SUVR | 15.40 (13.93) | 0.269 |
| Model 2 | DAT-AP | -5.85 (3.78) | 0.121 |
|  | DAT-PP | -1.94 (4.04) | 0.631 |
|  | DAT-AC | -11.19 (4.49) | 0.013 |
|  | DAT-PC | -10.63 (6.80) | 0.118 |
|  | FDR-PP | -8.12 (43.38) | 0.852 |
|  | FBB-SUVR | 22.84 (12.99) | 0.079 |
| Model 3 | DAT-AP | -5.83 (3.25) | 0.072 |
|  | DAT-PP | -3.14 (3.54) | 0.375 |
|  | DAT-AC | -8.96 (4.23) | 0.034 |
|  | DAT-PC | -7.11 (6.52) | 0.276 |
|  | FDR-PP | 9.11 (38.39) | 0.812 |
|  | FBB-SUVR | 10.85 (12.28) | 0.377 |

Results are based on general linear models for PC1 performed in DLB patients. Model 1 analyses were adjusted for age, sex, education, HTN, DM, DWMH, and PWMH. Model 2 analyses were further adjusted for UPDRS motor score, and Model 3 analyses were further adjusted for MMSE score from Model 1.

Key: DAT, dopamine transporter; DAT-AC, DAT uptake in the anterior caudate; DAT-AP, DAT uptake in the anterior putamen; DAT-PC, DAT uptake in the posterior caudate; DAT-PP, DAT uptake in the posterior putamen; DLB, dementia with Lewy bodies; DM, diabetes mellitus; DWMH, deep white matter hyperintensities; FBB-SUVR, ^18^F-Florbetaben standardized uptake value ratio; FDG, ^18^F-fluorodeoxyglucose; FDR-PP, FDG to DAT ratio in the posterior putamen; HTN, hypertension; MMSE, Mini-Mental State Examination; PC1, the first principal component of FDG subject residual profile; PWMH, periventricular WMH; UPDRS, Unified Parkinson’s Disease Rating Scale.

**Supplementary Table 3.** Comparison of imaging biomarkers and neuropsychological z scores between DLB patients with RBD and those without RBD

|  | DLB^RBD-^ (n = 23) | DLB^RBD+^ (n = 32) | P value |
| --- | --- | --- | --- |
| DAT-PP | 5.0 (1.2) | 5.0 (1.7) | 0.779 |
| DAT-AP | 5.8 (1.1) | 5.7 (1.9) | 0.698 |
| DAT-PC | 3.3 (0.8) | 3.5 (0.9) | 0.531 |
| DAT-AC | 4.6 (1.0) | 4.7 (1.5) | 0.848 |
| FDR-PP | 0.3 (0.1) | 0.4 (0.1) | 0.113 |
| PC1 | 48.7 (48.7) | 40.3 (39.6) | 0.456 |
| FBB-SUVR | 1.7 (0.4) | 1.5 (0.4) | 0.238 |
| Attention | -1.0 (1.2) | -0.6 (1.0) | 0.089 |
| Language | -1.9 (1.7) | -1.1 (1.6) | **0.025** |
| Visuospatial function | -3.1 (4.6) | -2.1 (2.4) | 0.253 |
| Memory function | -1.7 (0.9) | -1.2 (0.8) | **0.040** |
| Executive function | -1.9 (0.9) | -1.2 (0.9) | **0.004** |

Data are expressed in mean (SD). The *p* values are the result of general linear models controlling for age, sex, and education.

Key: DAT, dopamine transporter; DAT-AC, DAT uptake in the anterior caudate; DAT-AP, DAT uptake in the anterior putamen; DAT-PC, DAT uptake in the posterior caudate; DAT-PP, DAT uptake in the posterior putamen; DLB, dementia with Lewy bodies; FBB-SUVR, ^18^F-Florbetaben standardized uptake value ratio; FDG, ^18^F-fluorodeoxyglucose; FDR-PP, FDG to DAT ratio in the posterior putamen; PC1, the first principal component of FDG subject residual profile; RBD, rapid eye movement behavior disorder.

**Supplementary Table 4.** Association for imaging biomarkers with AI and absolute AI in DLB patients

|  | AI |  | Absolute AI |  |
| --- | --- | --- | --- | --- |
|  | Beta (SE) | P value | Beta (SE) | P value |
| DAT-PP | 0.33 (0.62) | 0.593 | -0.36 (0.45) | 0.424 |
| DAT-AP | 0.39 (0.56) | 0.498 | -0.72 (0.40) | 0.075 |
| DAT-PC | 0.87 (1.05) | 0.411 | -1.02 (0.75) | 0.179 |
| DAT-AC | 0.66 (0.69) | 0.345 | -0.98 (0.48) | **0.049** |
| FDR-PP | -2.39 (6.70) | 0.723 | 4.42 (4.79) | 0.362 |
| PC1 | -0.03 (002) | 0.158 | 0.05 (0.01) | **0.001** |
| FBB-SUVR | -0.04 (2.18) | 0.986 | 1.66 (1.56) | 0.292 |

Results are based on general linear models for AI or absolute AI after controlling for age, sex, education, HTN, DM, DWMH, and PWMH.

Key: AI, asymmetry index; DAT, dopamine transporter; DAT-AC, DAT uptake in the anterior caudate; DAT-AP, DAT uptake in the anterior putamen; DAT-PC, DAT uptake in the posterior caudate; DAT-PP, DAT uptake in the posterior putamen; DLB, dementia with Lewy bodies; DM, diabetes mellitus; DWMH, deep white matter hyperintensities; FBB-SUVR, ^18^F-Florbetaben standardized uptake value ratio; FDG, ^18^F-fluorodeoxyglucose; FDR-PP, FDG to DAT ratio in the posterior putamen; HTN, hypertension; PC1, the first principal component of FDG subject residual profile; PWMH, periventricular WMH.


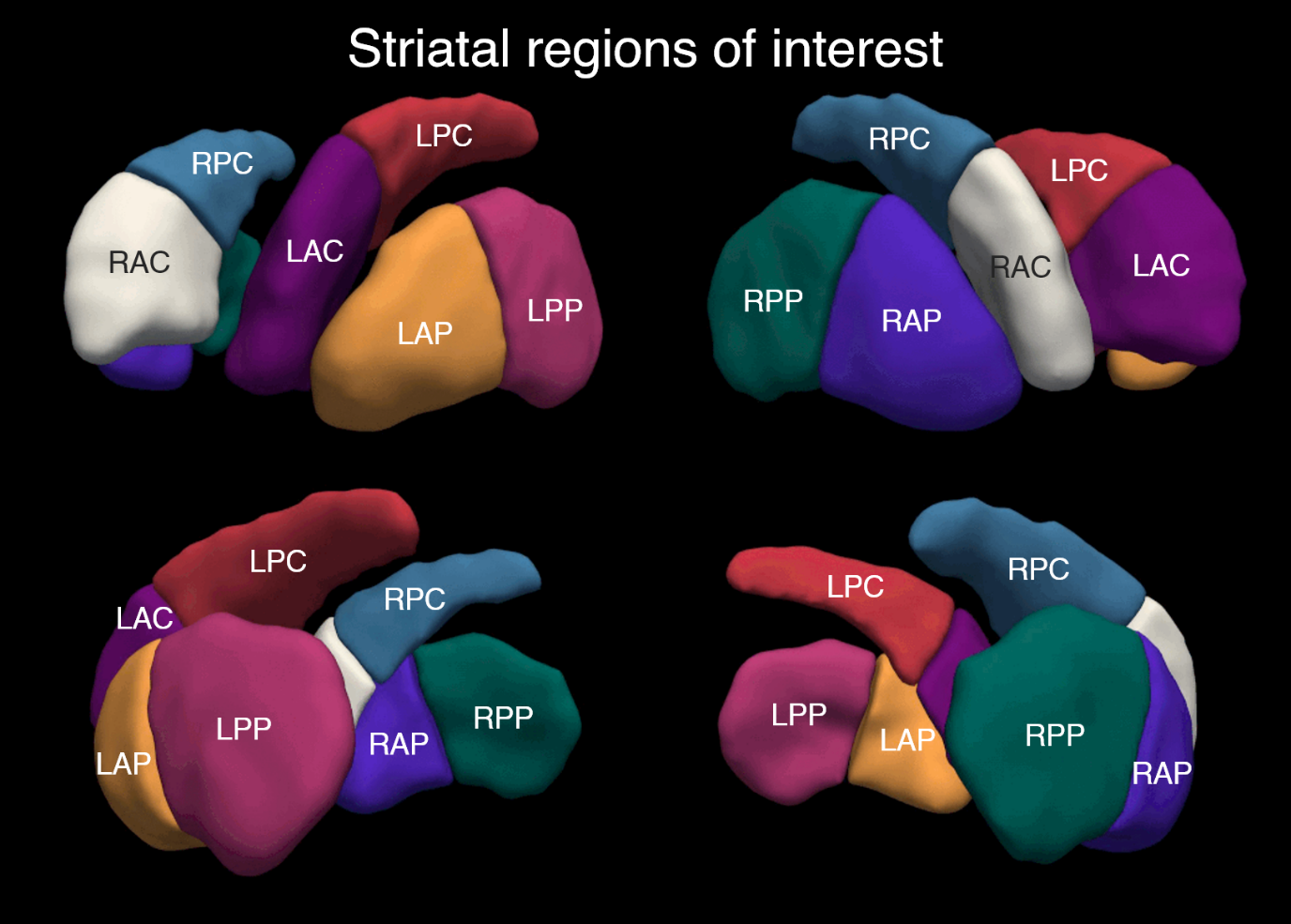


**Supplementary Figure 1.** **Striatal regions of interest**

Key: LAP, left anterior putamen; LPP, left posterior putamen; LAC, left anterior caudate; LPC, left posterior caudate; RAP, right anterior putamen; RPP, right posterior putamen; RAC, right anterior caudate; RPC, right posterior caudate.


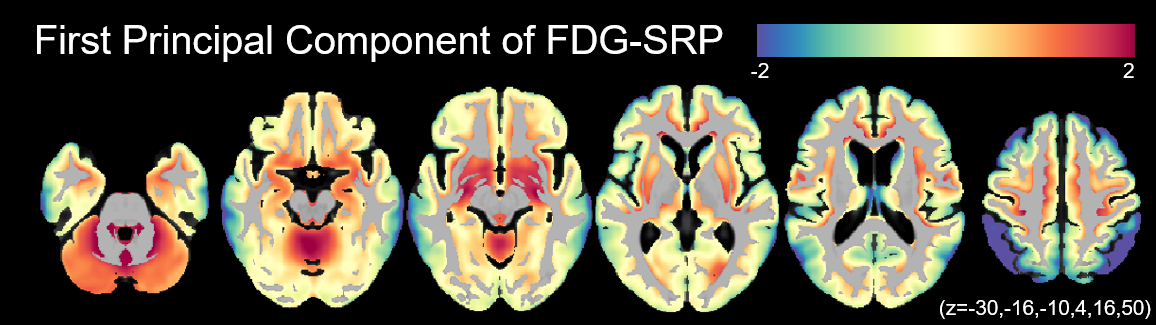


**Supplementary Figure 2.** The figure displays the first component of principal component analysis explaining 18.91% variance of the variability of the ^18^F-Fluorodeoxyglucose subject residual profile (FDG-SRP) within the gray matter brain mask. The axial planes are displayed in the Montreal Neurological Institute stereotaxic space in neurological convention (z=-30, -16, -10, 4, 16, 50).


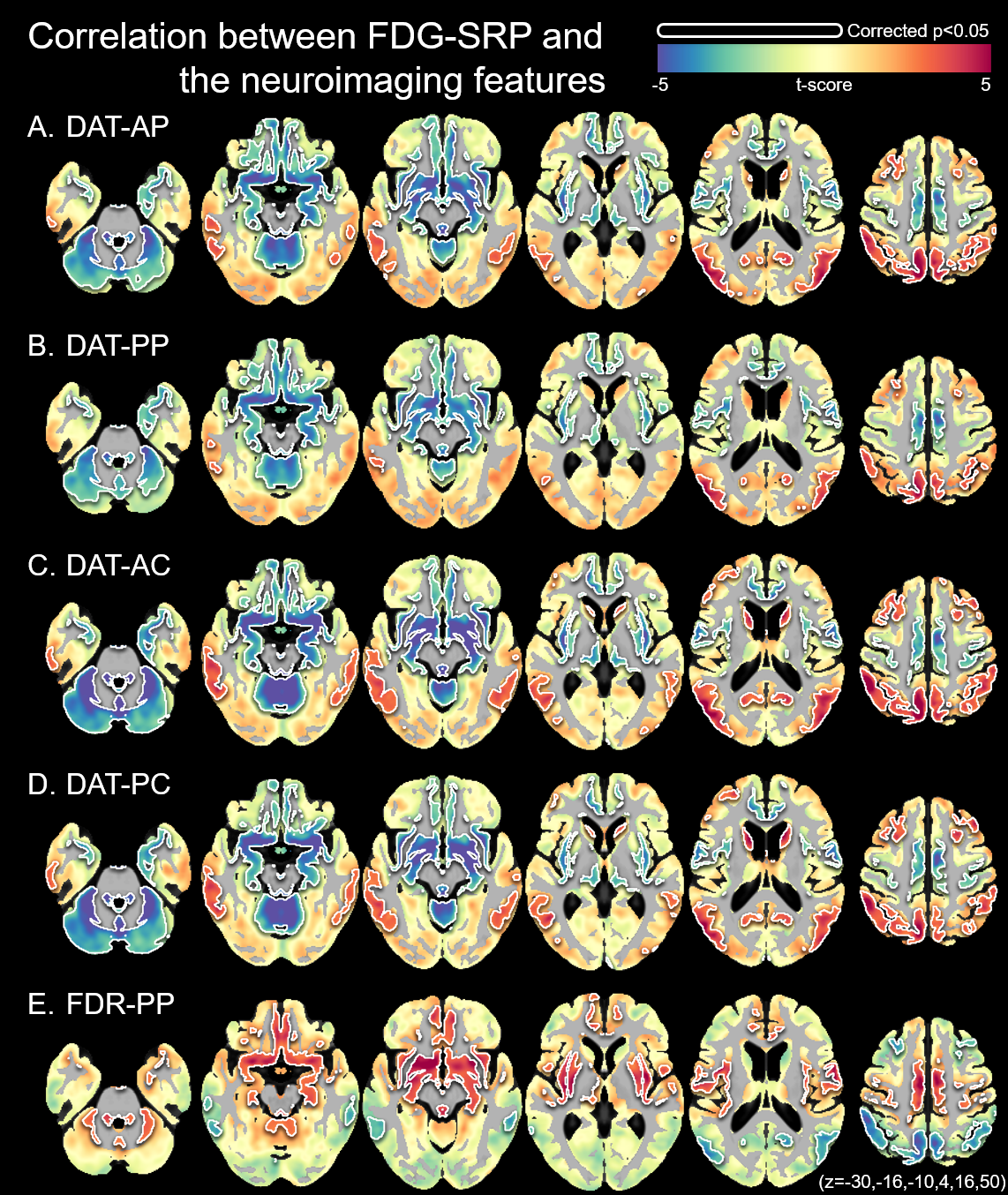


**Supplementary Figure 3.** Correlation between FDG-SRP and the dopamine transporter biomarkers. Results are based on general linear models controlling for age, sex, education, hypertension, diabetes mellitus, and deep and periventricular white matter hyperintensities. The color scale indicates t-values in the statistical analysis where red and blue color represent brain regions with positive and negative correlations, respectively. Areas bounded by a white line indicate brain regions where the correlation is significant after correcting for multiple comparisons (corrected p < 0.05, false discovery ratio). The axial planes are displayed on the Montreal Neurological Institute stereotaxic space (z = -30, -16, -10, 4, 16, 50).

Key: FDG- SRP, 18F-Fluorodeoxyglucose subject residual profile; DAT, dopamine transporter; DAT-AP, DAT uptake in the anterior putamen; DAT-PP, DAT uptake in the posterior putamen; DAT-PC, DAT uptake in the posterior caudate; DAT-AC, DAT uptake in the anterior caudate; FDR-PP, FDG/DAT in the posterior putamen.
